# *Candida albicans* Sap6 amyloid regions function in cellular aggregation and zinc binding, and contribute to zinc acquisition

**DOI:** 10.1038/s41598-017-03082-4

**Published:** 2017-06-06

**Authors:** Rohitashw Kumar, Christine Breindel, Darpan Saraswat, Paul J. Cullen, Mira Edgerton

**Affiliations:** 1 Department of Oral Biology, School of Dental Medicine, University at Buffalo, Buffalo, NY USA; 2 Department of Biological Sciences, University at Buffalo, Buffalo, NY USA

## Abstract

*Candida albicans* is an opportunistic fungal pathogen colonizing the oral cavity. *C*. *albicans* secreted aspartic protease Sap6 is important for virulence during oral candidiasis since it degrades host tissues to release nutrients and essential transition metals. We found that zinc specifically increased *C*. *albicans* autoaggregation induced by Sap6; and that Sap6 itself bound zinc ions. *In silico* analysis of Sap6 predicted four amyloidogenic regions that were synthesized as peptides (P1–P4). All peptides, as well as full length Sap6, demonstrated amyloid properties, and addition of zinc further increased amyloid formation. Disruption of amyloid regions by Congo red significantly reduced auotoaggregation. Deletion of *C*. *albicans* genes that control zinc acquisition in the *ZAP1* regulon, including zinc transporters (Pra1 and Zrt1) and other zinc-regulated surface proteins, resulted in lower autoaggregation and reduction of surface binding of Sap6. Cells with high expression of *PRA1* and *ZRT1* also showed increased Sap6-mediated autoaggregation. *C*. *albicans* ∆*sap6* deletion mutants failed to accumulate intracellular zinc comparable to ∆*zap1*, ∆*zrt1*, and ∆*pra1* cells. Thus Sap6 is a multi-functional molecule containing amyloid regions that promotes autoaggregation and zinc uptake, and may serve as an additional system for the community acquisition of zinc.

## Introduction

*Candida albicans* is commensal fungi that colonizes the human mucosal surfaces, including the oral cavity; and may become pathogenic in response to alterations in the host environment to cause oropharyngeal candidiasis (OPC). During OPC, the fungal burden on the tongue increases and *C*. *albicans* forms surface-localized plaques consisting of large aggregates of fungal cells loosely attached to the upper epithelial layers of the tongue mucosa^1, 2^. Histological observations showed these plaques contain aggregates of germinated *C*. *albicans* in which elongated filaments often penetrate into the underlying epithelium^3^.

Analyses of *C*. *albicans* genes expressed in fungal cells recovered from mouse tongue plaques in OPC identified two members of the secreted aspartic proteinase (Sap) family, *SAP6* and *SAP5*, to be among the most highly expressed genes^4^. Furthermore, a *C*. *albicans* strain over-expressing *SAP6* was hyper-virulent in murine OPC^3^. Sap6 is primarily secreted upon fungal germination and is the second most abundant protein secreted into the medium by hyphal cells^5^. The best-characterized function of *C*. *albicans* Saps is proteolytic digestion of host tissues to release nutrients such as oligopeptides and amino acids^6, 7^. However, we found that Sap6 and Sap5 also have a non-proteolytic function in mediating cell-to-cell aggregation (autoaggregation) that promotes *C*. *albicans* adhesion to both other cells and host epithelium^3^. Saps may also have additional functions in nutrient acquisition by bringing yeast cells together to share released host peptides and other essential divalent metals. Thus, Sap5 and Sap6 may function as community-organizing molecules to promote beneficial social networks between fungal cells to allow sharing of nutrients obtained by proteolytic digestion of host tissues.

Nutritional immunity is one facet of host defense against microorganisms that functions by withholding essential metals including zinc, iron and copper in a sequestered form not readily available to invading pathogens^8, 9^. For example, human calprotectin sequesters zinc and thus inhibits fungal growth by reducing localized zinc levels at mucosal cell surfaces^10, 11^. Zinc is the second most abundant transition metal in the human body, and like iron, its availability is tightly regulated^12^. Zinc is a cofactor for all classes of enzymes in most living organisms and stabilizes Zn-finger domains, a function that is especially important in eukaryotes^12, 13^. As *C*. *albicans* is very sensitive to zinc starvation, it has developed strategies to efficiently forage zinc under zinc limiting conditions to overcome nutritional immunity^14^.

*C*. *albicans* Zap1/Csr1 is a transcription factor and an ortholog of *S*. *cerevisiae* Zap1 (Zinc-responsive activator protein), that has a critical role in zinc metabolism. In *S*. *cerevisiae*, Zap1 controls the expression of zinc transporters and other zinc-regulated genes dependent upon zinc availability^15^. Similarly, the *C*. *albicans ZAP1* regulon tightly regulates zinc acquisition genes *ZRT1*, *ZRT2*, and *PRA1* ^16^. *C*. *albicans* cell surface zinc transporters Zrt1 and Zrt2 and secreted Pra1 (pH-regulated antigen 1) all contribute to zinc acquisition via a “zincophore” system similar to iron-carrying siderophores. Structural studies of Pra1 suggested the presence of two zinc-binding coordination sites and several putative zinc binding sites including one metal binding HRXXH motif^14^. Secreted Pra1 was shown to complex back with the extracellular regions of Zrt1, perhaps through zinc bridges to allow zinc uptake^14, 17^.

Recent studies showed that *C*. *albicans* genes involved in metal acquisition were among up-regulated genes during *Candida* infection (both OPC and invasive infection); including *ZRT1*, *PRA1*, as well as *SAP5* and *SAP6* ^4, 18^. The co-expression of *C*. *albicans* hyphal specific Saps along with genes related to zinc acquisition at different infection niches suggests they might have complementary functional roles^18^. Finkel *et al*. showed that *ZAP1*, along with other transcription factors, were part of a complex regulatory network governing the expression of cell surface proteins (including Sap5 and Sap6) involved in cell adherence^16^.

We previously discovered that secreted Sap6 binds to the *C*. *albicans* surface to promote cell aggregation; and that Sap6 RGD motifs were partly responsible for this aggregation. Deletion of Sap6 RGD motifs reduced aggregation by 40%, showing that additional Sap6 components contributed to autoaggregation^3^. We hypothesized that additional Sap6 aggregation motifs might be similar to those of *C*. *albicans* Als5 protein that contains highly conserved functional amyloidogenic regions that strengthen cell-to-cell binding and substrate adhesion^19, 20^. Many bacterial and fungal cell surfaces utilize functional amyloids to adhere cells to one another, to substrates or to host cells. In many cases, functional amyloids initiate cell-cell aggregation resulting in micro-colonies and biofilms during infection^21^. Unlike human brain amyloids that form large β-sheet fiber aggregates, it is thought that *C*. *albicans* Als5 amyloids consist of much smaller β-strand ribbons that form “nanodomains” or “patches” at the cell surface^19, 22, 23^. These amyloid nanodomains mediate cell-to-cell binding, as shown by the disruption of *C*. *albicans* aggregates by addition of the anti-amyloid dye Thioflavin T. It is also intriguing that amyloids, including human brain amyloids and the S100 family of zinc sequestering proteins, bind zinc and other divalent metals^24–26^.

Based upon the pronounced aggregative phenotype of Sap6, both *in vitro* and *in vivo*, as well as its co-expression with zinc acquisition genes in *C*. *albicans*, we hypothesized that *C*. *albicans* Sap6 may contain amyloid domains that mediate cell-to-cell aggregation and zinc binding. We further examined whether Sap6 could bind with other *C*. *albicans* cell surface proteins that are more highly expressed during OPC for cellular aggregation, and whether this aggregation had functional significance in promoting zinc accumulation. We show here for the first time that amyloidogenic regions within Sap6 are responsible for autoaggregation as well as zinc binding; and that Sap6 contributes to zinc uptake in fungal communities.

## Results

### Extracellular zinc increases cellular aggregation through its binding to Sap6

**Figure 1:**
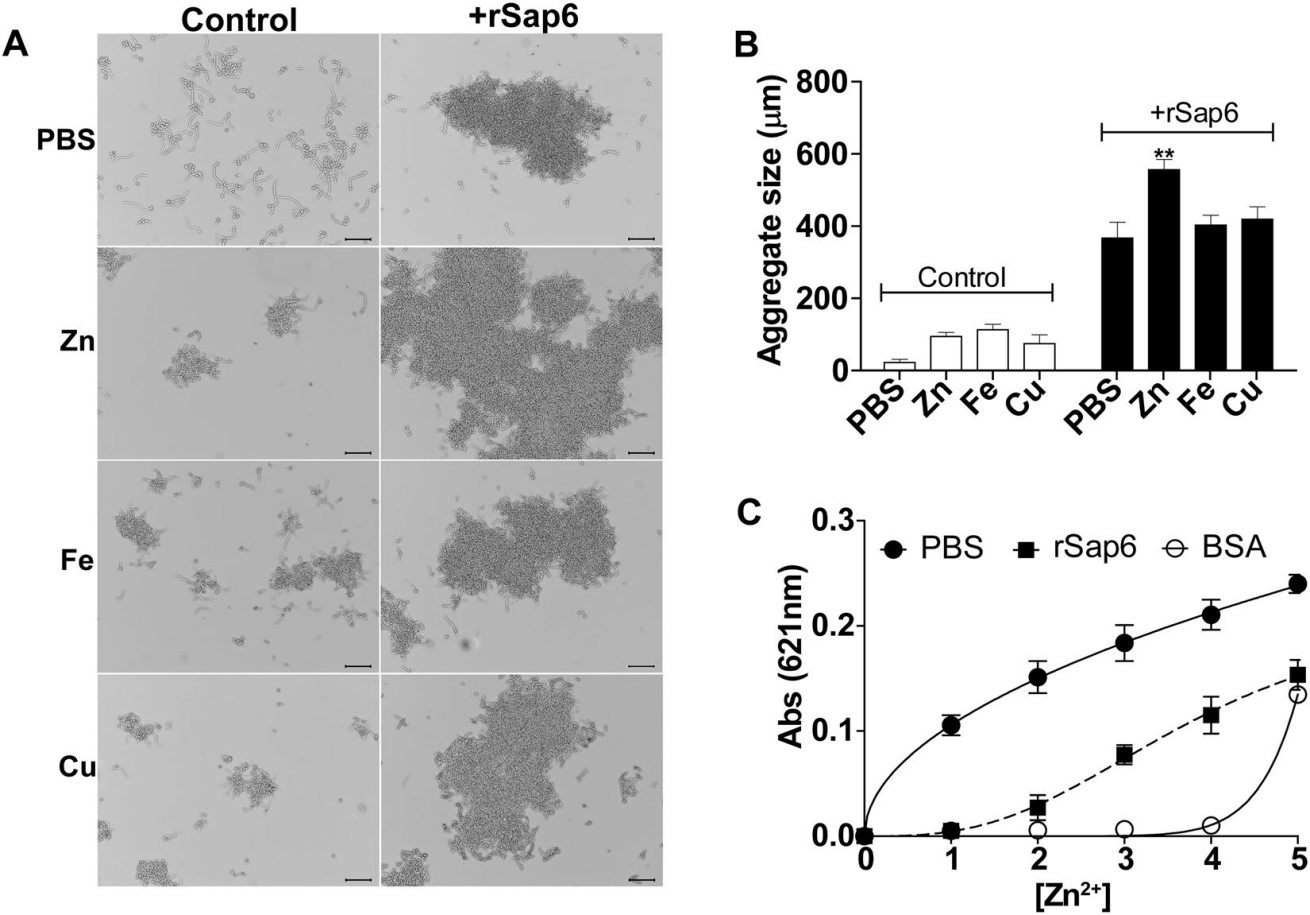
Sap6 preferably interacts with zinc to increase cellular adhesion. (**A**) Wild type (WT) cells were germinated for 3 h at 37 °C and then incubated either with metal alone or metal pre-incubated with rSap6 for 15 min and observed microscopically. Addition of ZnCl_2_ (10 µM), FeCl_3_ (10 µM) and CuSO_4_ (10 µM) as metal control induced small aggregates compared to PBS. The aggregates were observed and documented at 10X. (**B**) ImageJ software was used to analyze aggregate size. Germinated cells incubated with metal control (ZnCl_2_, FeCl_3_ and CuSO_4_) induced aggregation compared to PBS (white bars). The aggregate size after addition of Zn-rSap6 complex was significantly increased whereas there was no significant change in aggregate size after adding Fe or Cu-rSap6 complex (black bars). The bars show mean ± SD of at least three independent experiments. The P-value was ≤0.01 and was calculated using one-way ANOVA. (**C**) Zincon (20 µM) (PBS) alone, or Zincon mixed with rSap6 or bovine serum albumin (BSA) (2:1) in 75 mM HEPES, 100 mM NaCl, pH 7.4 were titrated against Zn^2+^. Addition of one to five equivalents of Zn^2+^ proportionally decreased absorbance in Zincon + Sap6 suggesting up to five Zn^2+^ affinity sites in Sap6; while BSA served as positive control and showed a minimum of four zinc binding sites. Measurements were performed at three independent time points in triplicate and the mean ± SD are shown.

We have previously shown that *C*. *albicans* cells over-expressing Sap6 had enhanced cellular aggregates in plaques from mice tongues during OPC^3^; and others showed up-regulation of *SAP4-6* along with genes involved in divalent metal homeostasis (especially zinc and iron) in OPC^4^. We hypothesized that *C*. *albicans* Sap6 may also participate in binding divalent metal ions because of its co-expression with zinc and iron homeostasis genes, along with cell aggregation, during OPC. Therefore, we directly tested whether zinc, iron and copper were able to induce changes in Sap6-mediated cellular aggregation. Wild type *C*. *albicans* cells were germinated at 37 °C in GlcNAc medium (3 h), washed twice with PBS and incubated for 15 min with purified rSap6 (10 µM) to induce aggregation (Fig. 1A right panel; WT). Under these standard conditions, germinated cells do not form large aggregates unless Sap6 is added. However, addition of divalent ions alone (zinc, 10 µM ZnCl_2_; iron, 10 µM FeCl_3_; copper, 10 µM CuSO_4_) to germinated cells for 15 min at 37 °C resulted in an increase in aggregation for all metals (Fig. 1A, left panel). This result shows that divalent metal ions induce hyphal aggregation. We also found that addition of rSap6 caused a specific aggregation response to zinc (Fig. 1A and B, right panel). Pre-incubation of rSap6 with zinc significantly increased the average aggregate size from 366 µm to 556 µm (Fig. 1B), while rSap6 pre-incubated with iron or copper had no significant change in aggregate size. Thus, among these divalent metals tested, zinc induces a specific Sap6-dependent aggregation response suggesting binding between Zn^2+^ and Sap6.

To directly test binding between Zn and Sap6, titration of zinc binding was done using the zinc chelator Zincon. Zincon is a colorimetric low affinity Zn^2+^ indicator with a dissociation constant of (K_D_ ≈10 µM)^27^. Titration of a 1:2 mixture of rSap6 and Zincon with increasing amounts of Zn^2+^ ions showed a proportional decrease in absorbance. Specifically, addition of one equivalent of Zn^2+^ ion reduced absorbance, which indicates that Sap6 binds to one zinc ion with higher affinity than Zincon (Fig. 1C). Addition of two to five equivalents of Zn^2+^ proportionally decreased absorbance suggesting the presence of up to four additional lower affinity Zn binding sites in Sap6. As expected for a positive control, BSA (that has amyloid structure and at least four zinc binding domains) strongly bound zinc (Fig. 1C, open circles). Using a K_D_ value for Zn^2+^-Zincon of 10 µM we calculated an apparent binding constant (K_app_) for Sap6 across all Zn^2+^ binding sites to be K_app_ = 7.2 µM; showing that zinc strongly binds with Sap6 protein.

### Sap6 contains predicted zinc-binding sites and amyloidogenic regions

**Figure 2:**
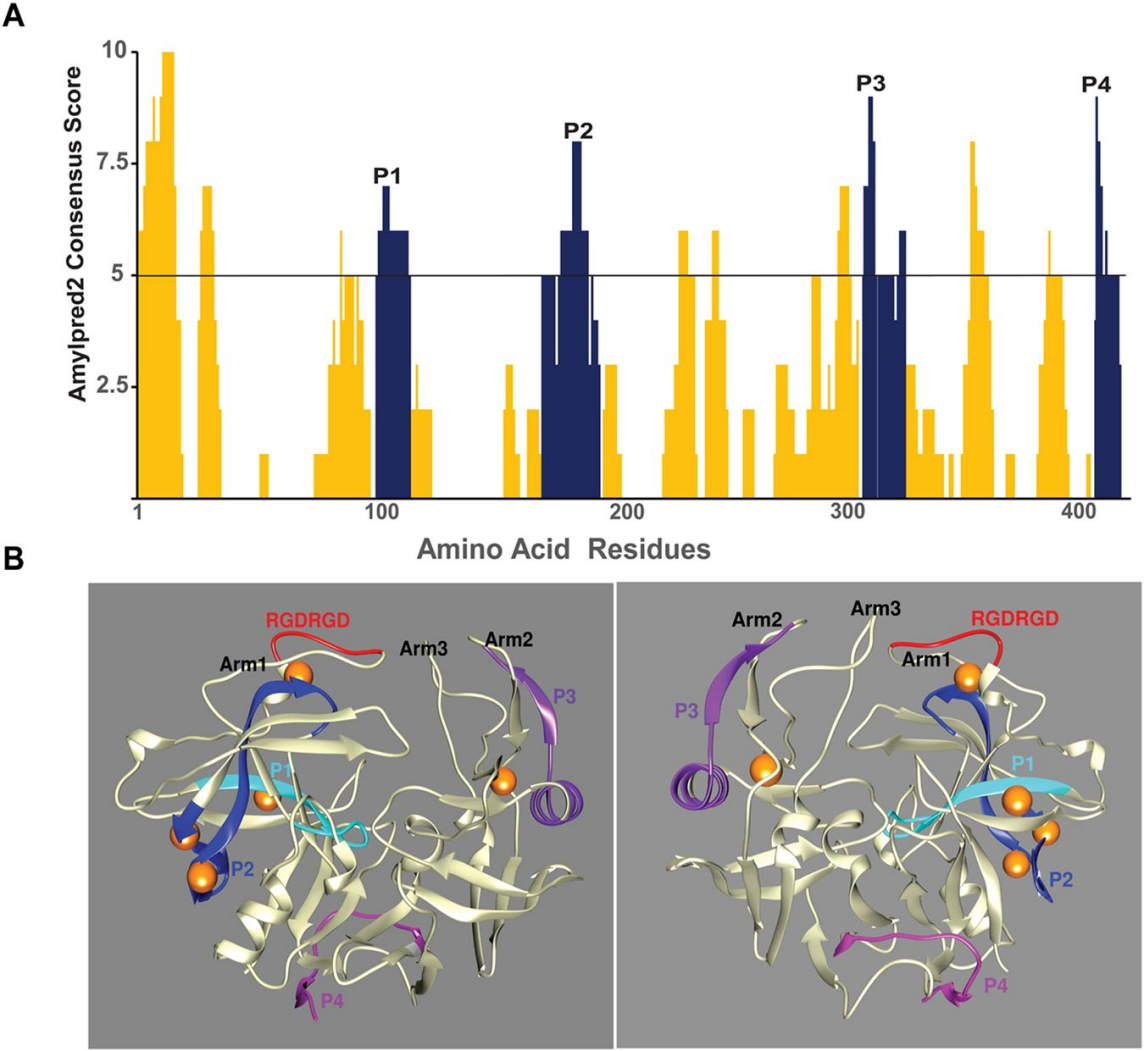
Sap6 contains amyloidogenic regions and zinc binding sites. (**A**) Sap6 primary structure is plotted with the likelihood of amyloidogenic structures using AmylPred2 prediction algorithms. Regions in which more than 5 out of 11 algorithms predict amyloid structure are shown in blue color bars and are designated as P1 (K^102^LSVIVDTGSS^112^), P2 (Q^172^DTVGIGGASVKNQLFANVWSTSA^195^), P3 (A^307^RSIIYALGGQVHFD^321^), and P4 (A^404^QVKYTSESNIVAIN^416^). (**B**) A three-dimensional model of Sap6 was constructed using Phyre2 software. Zinc binding sites were predicted using both amino acid sequence and three-dimensional structure of Sap6 using Ioncom server and Metal Ion-Binding (MIB) server^47, 48^. All predicted amyloid regions (P1 turquoise, P2 blue, P3 purple, P4 magenta) contain at least one beta strand and are surface localized. Two zinc-binding sites were found in the P2 region (zinc is shown with gold spheres); and a third predicted site is within the P1 region, while two other zinc-binding sites lie within non-amyloid regions. The surface-localized RGD binding domain (red) lies adjacent to the P2 region as well as a zinc-binding site within arm 1.

Sap3 contains putative amyloidogenic regions, and zinc ions are required for formation of amyloid structure^20, 21^. We hypothesized that Sap6 contains amyloidogenic regions that may account for the zinc-mediated cellular aggregation. Using AmylPred2 software (that identifies amyloidogenic regions based on a consensus score of 11 computational tools), we identified four putative amyloidogenic regions within *C*. *albicans* Sap6 with a consensus score ≥5 (Fig. 2A, blue peaks). These microdomains were designated P1 (K^102^LSVIVDTGSS^112^), P2 (Q^172^DTVGIGGASVKNQLFANVWSTSA^195^), P3 (A^307^RSIIYALGGQVHFD^321^), and P4 (A^404^QVKYTSESNIVAIN^416^). Next, we generated a predicted three-dimensional structure of Sap6 using Phyre2 software in which the four computationally identified amyloidogenic regions (P1–P4) were mapped within the three-dimensional structure (Fig. 2B). Our structural model of Sap6 showed that major portions of these four predicted amyloidogenic regions are surface exposed, in line with the idea that they may facilitate functional interactions. P1 and P2 are in close proximity to Arm1 (Fig. 2B, turquoise and blue respectively), which contains the sole R^128^GDRGD^133^domain (Fig. 2B, red) that is involved in cellular aggregation and binding to host surfaces^3, 28^. This suggests cooperative interactions between the RGD domain and P1 and/or P2. The P2 region contains three beta strands, which is a typical antiparallel-type amyloidogenic motif ^23^. P3 (Fig. 2B, purple) is found in Arm2 and contains both an alpha helix and beta strand. P4 (Fig. 2B, magenta) is located in the region connecting the distal arm of Sap6 and contains two beta strands.

Three independent tests were used to predict zinc-binding sites in Sap6. We chose a coordinate distance of <3.5 Å between metal and target amino acid. Two potential zinc-binding sites occur in the P2 region. One is at position T^193^ and the other is at position D^173^ (Fig. 2B). A third predicted site occurred in on P1 at position D^108^. Two other zinc-binding sites were also identified in non-amyloid regions. One is at the enzyme active site at position T^297^, which might suggest that Sap6 requires zinc as a cofactor for its enzymatic activity.

### Amyloid regions in Sap6 correspond to increased cell aggregation and are zinc-dependent

**Figure 3:**
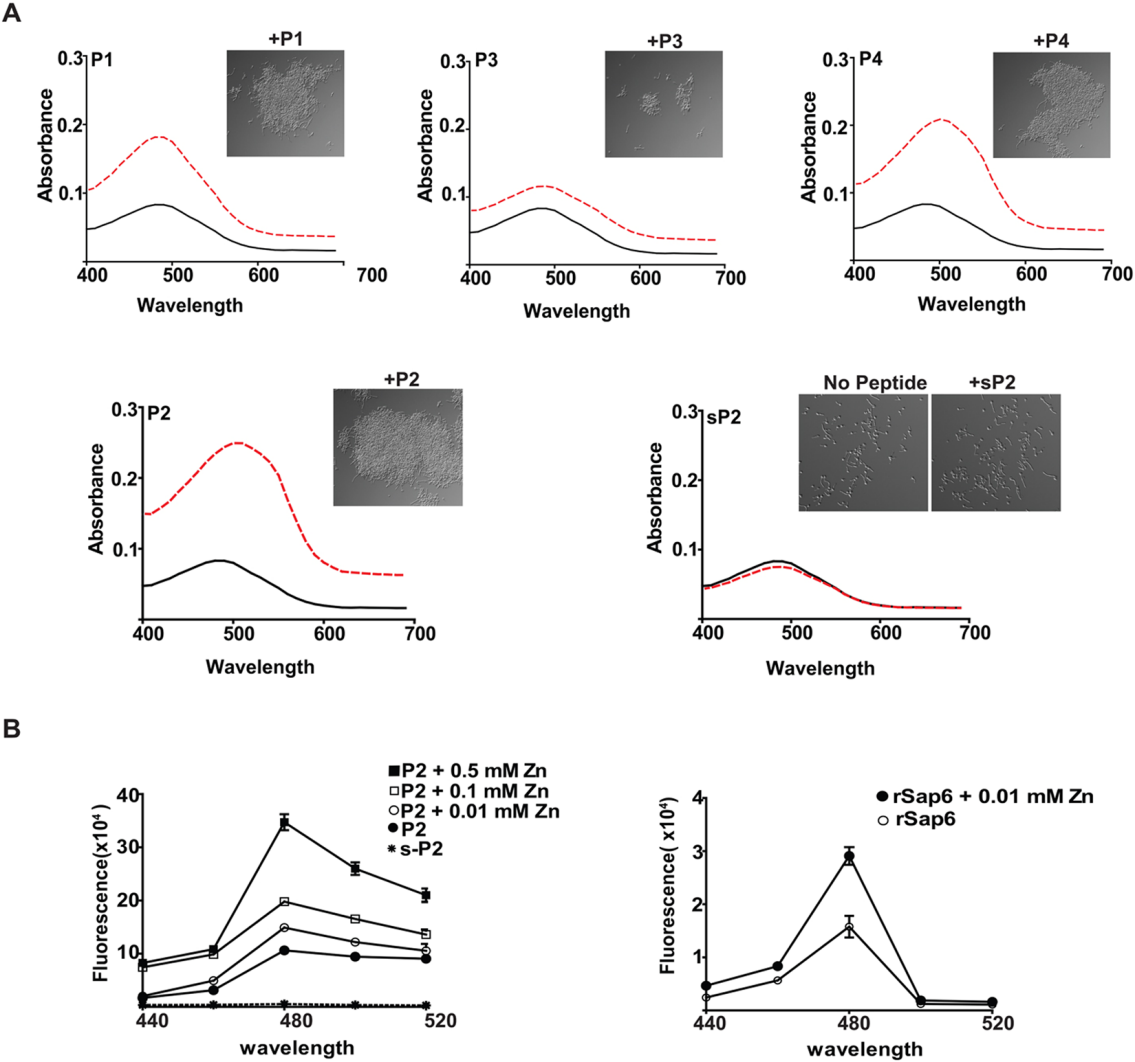
Sap6 amyloid regions induce fungal cell aggregation proportionally with the degree of amyloid structure shown by Congo red binding, and zinc further increases amyloid structure. (**A**) Amyloid structure of peptides P1–P4 and a scrambled P2 peptide (sP2) was evaluated by their affinity for Congo red that specifically binds to amyloid structures. Absorbance spectra (400–700 nm) of Congo red alone (solid black lines) or with peptides P1–P4 (dashed red lines) were measured. Autoaggregation of germinated *C*. *albicans* was observed microscopically following 15 min incubation with each peptide. Cell aggregation was the largest for P2, with moderate autoaggregation with P4 and P1, while P3 induced very little aggregation. Amyloid structure (as shown by amount of Condo red absorbance) was largest for P2, followed by P1 and P4, while P3 showed the least absorbance. The scrambled peptide sP2 did not form aggregates nor show Congo red absorbance. (**B**) Thioflavin T (ThT) binding with amyloid structures was measured (440 nm–520 nm) with peptide P2 and sP2 (10 µM) (left panel) or rSap6 (right panel) following pre-incubation with ZnCl_2_ (0–0.5 mM) for 4 h. ThT fluorescence was maximal at 480 nm (showing the presence of amyloid structure) for both P2 and full length rSap6, but the scrambled P2 peptide had no evidence of amyloid structure as shown by the lack of ThT absorbance. Addition of zinc to both P2 and rSap6 increased amyloid structure as shown by the increased fluorescence. Data are the mean ±SD for three independent experiments.

To test whether Sap6 forms amyloid structures, P1, P2, P3 and P4 peptides were synthesized and compared to a control scrambled P2 peptide (sP2) for their ability to induce aggregation in germinated *C*. *albicans* cells. We also tested for amyloid structure by affinity for Congo red, which binds to amyloid structures^20, 29^. All four peptides showed Congo red absorbance (Fig. 3A, red dotted lines) compared to the scrambled sP2 peptide or peptide alone (Fig. 3A, black lines). Congo red affinity for each peptide corresponded to the degree of *C*. *albicans* aggregation. P2 peptide induced the highest aggregative phenotype when added to germinated fungal cells, followed by P1, P3 and P4 peptides (shown in panel inserts). Thus, regions of Sap6 with amyloid properties can induce fungal aggregation.

Since amyloids interact with metal ions, particularly zinc and copper, to increase amyloid aggregation^24, 25^, we expected that the P1–P4 regions of Sap6 would show a similar increase in amyloid structure in the presence of zinc. For these experiments, ThioflavinT (ThT) was used as a dye to assess the level of amyloid structure in the presence of zinc, because metals interfere with Congo red spectra. Peptide P2 showed an increase in fluorescence upon addition of ThT, while the scrambled P2 peptide (sP2) that served as a negative control showed no change in fluorescence (Fig. 3B). The addition of zinc caused a dose dependent increase in P2 fluorescence, indicating that zinc functions to increase the amyloid structure of the P2 region (Fig. 3B left panel). Full-length Sap6 protein also showed ThT fluorescence that was increased by the addition of zinc (Fig. 3B right panel). Thus, Sap6 contains surface exposed regions whose amyloid properties are increased in the presence of zinc.

### RGD and amyloid regions together contribute to Sap6 aggregation

**Figure 4:**
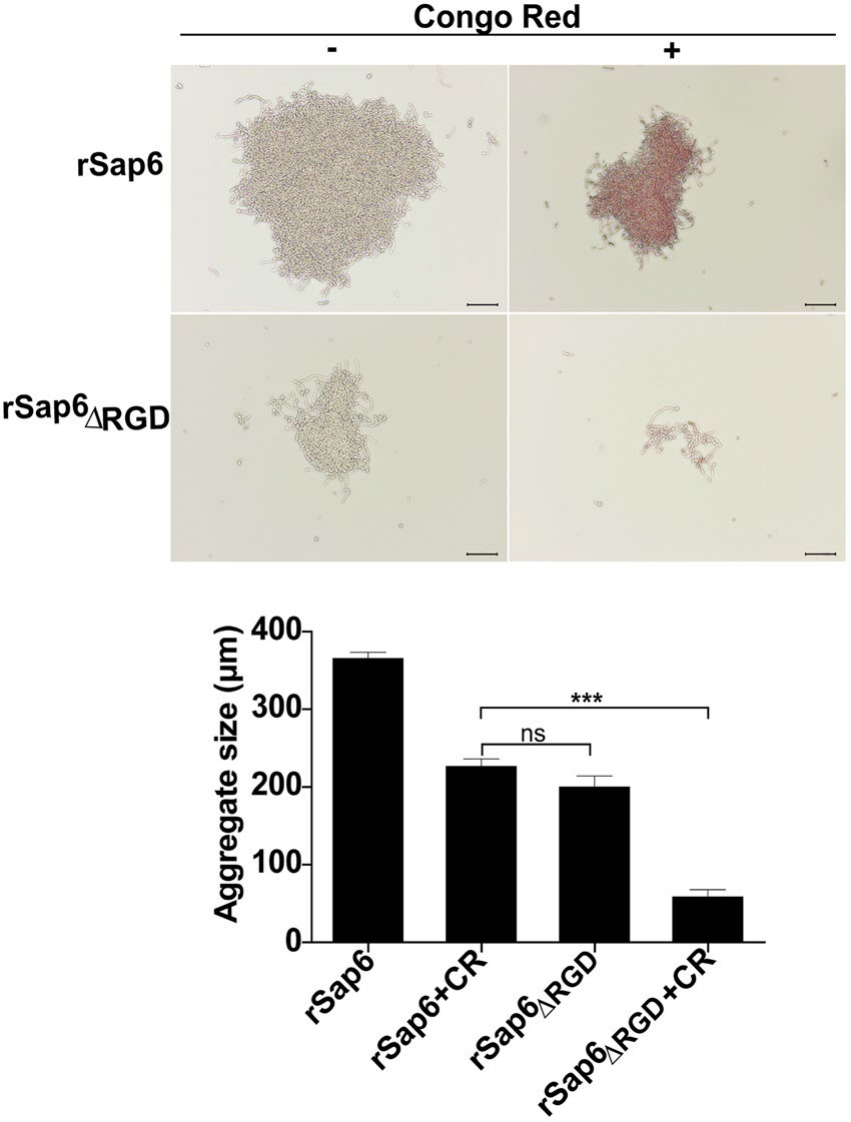
Amyloid regions and RGD binding sites in Sap6 both contribute to self-aggregation. Geminated *C*. *albicans* cells were incubated in the presence of Congo red (30 µM) along with rSap6 or rSap6_ΔRGD_ (10 µM) to allow Congo red binding to disrupt amyloid structure needed for autoaggregation. Average aggregate size was determined from 20 independent fields at 10X magnification using ImageJ software. Congo red significantly (P ≤ 0.01) decreased rSap6 aggregate size by nearly 45%, showing that amyloid structures account for nearly half of autoaggregation. Another 45% of aggregation was due to RGD sites as shown by the 90% reduction in aggregate size of rSap6_ΔRGD_ in the presence of Congo red. A small (10%) proportion of aggregation is independent of either amyloid regions or RGD. Mean ± SD of three independent experiments performed in triplicate are shown.

We previously showed that RGD motifs in Sap6 play a role in cellular aggregation^3^. To determine the relative contributions of amyloid regions and the RGD motifs in aggregation, we examined aggregation in the presence of Congo red. Congo red disrupted aggregate structure and reduced cellular aggregate size by 40% (Fig. 4, upper panel). We hypothesized that the remaining aggregation seen in cells in Congo red was due to its RGD region. As predicted, aggregation of cells induced by Sap6_ΔRGD_ protein were reduced by 45%, and addition of Congo red further reduced aggregation by 85% (Fig. 4, lower panel). Thus, *C*. *albicans* aggregation is mediated by Sap6 amyloid regions and its RGD domain.

### Genes regulated by *ZAP1* are most crucial for Sap6 for cellular aggregation

**Figure 5:**
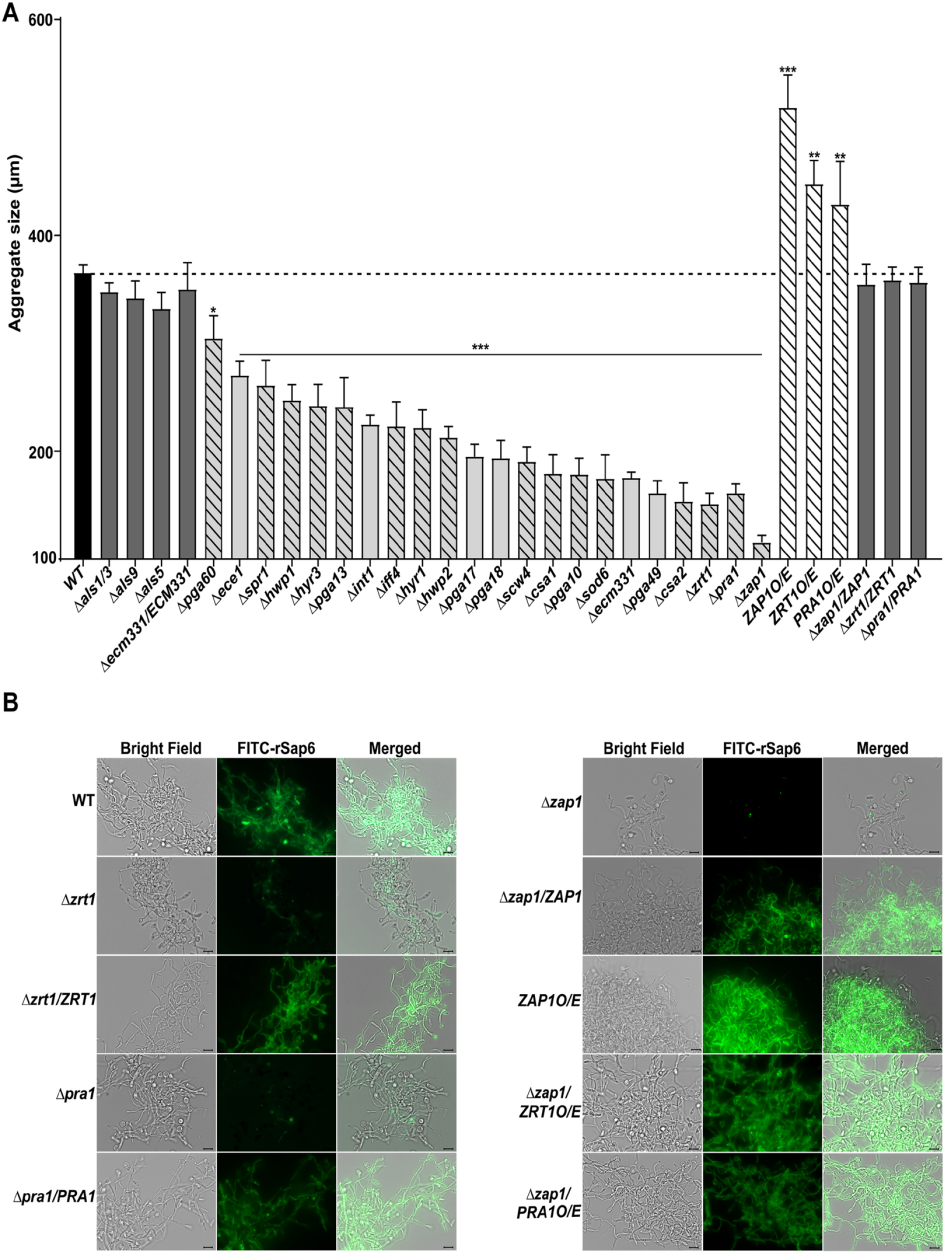
Genes regulated by *ZAP1* are required for Sap6 induced autoaggregation and cell surface binding. (**A**) Geminated *C*. *albicans* (WT CAI4 and deletion mutants) were incubated with rSap6 (10 µM) for 15 min and average aggregate size was determined from 20 independent fields at 10X magnification using ImageJ software. None of the *C*. *albicans ALS* deletion mutants had aggregation defects (black bars), showing that Als proteins are not involved in autoaggregation. The *C*. *albicans ZAP1* deletion mutant had the largest aggregation defect (aggregates were reduced by 90%) whereas *ZAP1* complemention reversed the aggregation defect. Several other zinc acquisition and homeostasis mutants (*PRA1*, *ZRT1*, *SOD6*) showed aggregation reduced by more than 50%. Most of the genes whose deletion resulted in a significant (***P < 0.01) decrease in aggregate size are regulated by *ZAP1* (hatched bars), although some (grey bars) are not known to be *ZAP1* regulated. Mean ± SD of three independent experiments for each strain are shown. (**B**) *C.albicans* CAI4, *Δzap1*, *Δzrt1* and Δ*pra1* deletion mutants, *Δzap1*/*ZAP1*, *Δzrt1*/*ZRT1* and Δ*pra1*/*PRA1* complementation strains and *ZAP1O*/*E*, *ZRT1O*/*E* and *PRA1O*/*E* over-expressing strains were germinated and incubated with FITC-labeled rSap6 (F-Sap6) for 1 h. After washing, cell surface binding of F-rSap6 was documented microscopically at 63X. Cell surface binding of F-Sap6 to Δ*zap1*, Δ*zrt1* and Δ*pra1* deletion strains was reduced compared with wild-type cells, while overexpression strains *ZAP1O*/*E*, *ZRT1O*/*E* and *PRA1O*/*E* showed increased cell surface binding of F-Sap6. Left panel, bright field; Center panel, FITC; and Right panel, merged bright field and FITC images.

To define cell surface binding partners for Sap6-dependent aggregation, we examined a panel of *C*. *albicans* deletion mutants for genes highly expressed during OPC and invasive candidiasis including those genes involved in metal homeostasis and adhesion^4^. Among 78 available mutants screened, 34 deletion strains (including 25 cell surface genes shown as bar graph) showed a reduction in aggregation. Surprisingly, cells lacking the major *C*. *albicans* adhesion proteins (including Als1, Als3, Als5 and Als9)^22^ did not show a significant change, suggesting that these adhesins are not involved in Sap6-mediated aggregation (Fig. 5A). By comparison, mutants in genes encoding other hyphal cell surface proteins including *PGA13*, *IFF4*, *SPR1*, *HYR1* and *HYR3*, hyphal specific *ECE1*, and major hyphal cell wall proteins *HWP1*, *HWP2* and *INT1* (with known functions in cell-to-cell adhesion and host attachment)^30, 31^ resulted in 20–40% reduction of Sap6 aggregate size. Even higher reduction in Sap6 aggregation (>40%) was found in mutants lacking genes encoding hyphal GPI-anchored cell wall proteins involved in cell surface adhesion and biofilm formation (*PGA17*, *PGA18*, *PGA49* and *ECM331*). Interestingly, the highest reduction in aggregate size (50–59%) was seen in mutants lacking zinc binding and homeostasis genes *SOD6*, *ZRT1* and *PRA1* along with genes involved in heme/hemoglobin iron utilization acquisition (*PGA10*, *CSA1* and *CSA2*). Interestingly, cells lacking the transcription factor *ZAP1* (a master zinc-responsive and cell adherence transcription factor)^16^ had the highest reduction in Sap6-mediated aggregate size, and this was reversed by *ZAP1* complementation (Fig. 5A). We observed that mutants with the largest defects in aggregation were those with *ZAP1* regulated genes (hatched bars, Fig. 5A), including genes encoding cell wall RGD binding proteins (*INT1* and *IFF4*).

To directly test whether the expression of *ZAP1* regulated genes affect Sap6 binding, FITC-labeled rSap6 (F-Sap6) was added to germinated *Δzap1* and *Δzrt1* cells, as well as in cells over-expressing *ZAP1* (*ZAP1O*/*E*), *ZRT1* (*ZRT1O*/*E*) in a *Δzap1* background, then the binding to Sap6 and size of aggregation was measured. Aggregates of the both the strains *Δzap1* and Δ*zrt1* strain bound substantially less F-Sap6 compared with the wild type strain (and was accompanied by smaller aggregation), while *ZAP1O*/*E* and *ZRT1O*/*E* strain aggregates were larger and had robust binding of F-Sap6 (Fig. 5A and B). We also evaluated surface binding of F-rSap6 in cells with deletion and overexpression of *PRA1*, another *ZAP1* regulated gene encoding a surface zinc-binding protein. Deletion of *PRA1* resulted in reduced surface binding of F-rSap6, while cells overexpressing Pra1 (*PRA1O*/*E*) in a *Δzap1* background showed restored cell surface binding of F-rSap6 (Fig. 5B). The complement strains *∆zap1*/*ZAP1*, *∆zrt1*/*ZRT1 and ∆pra1*/*PRA1* also showed rSap6 binding similar to wild type cells. Thus the presence of both Zrt1 or Pra1 mediate Sap6 surface binding and cellular aggregation; and suggests that other *ZAP1* regulated genes likely play a role on Sap6 autoaggregation.

### Increased expression of *PRA1* and *ZRT1* by zinc limitation increase aggregation

**Figure 6:**
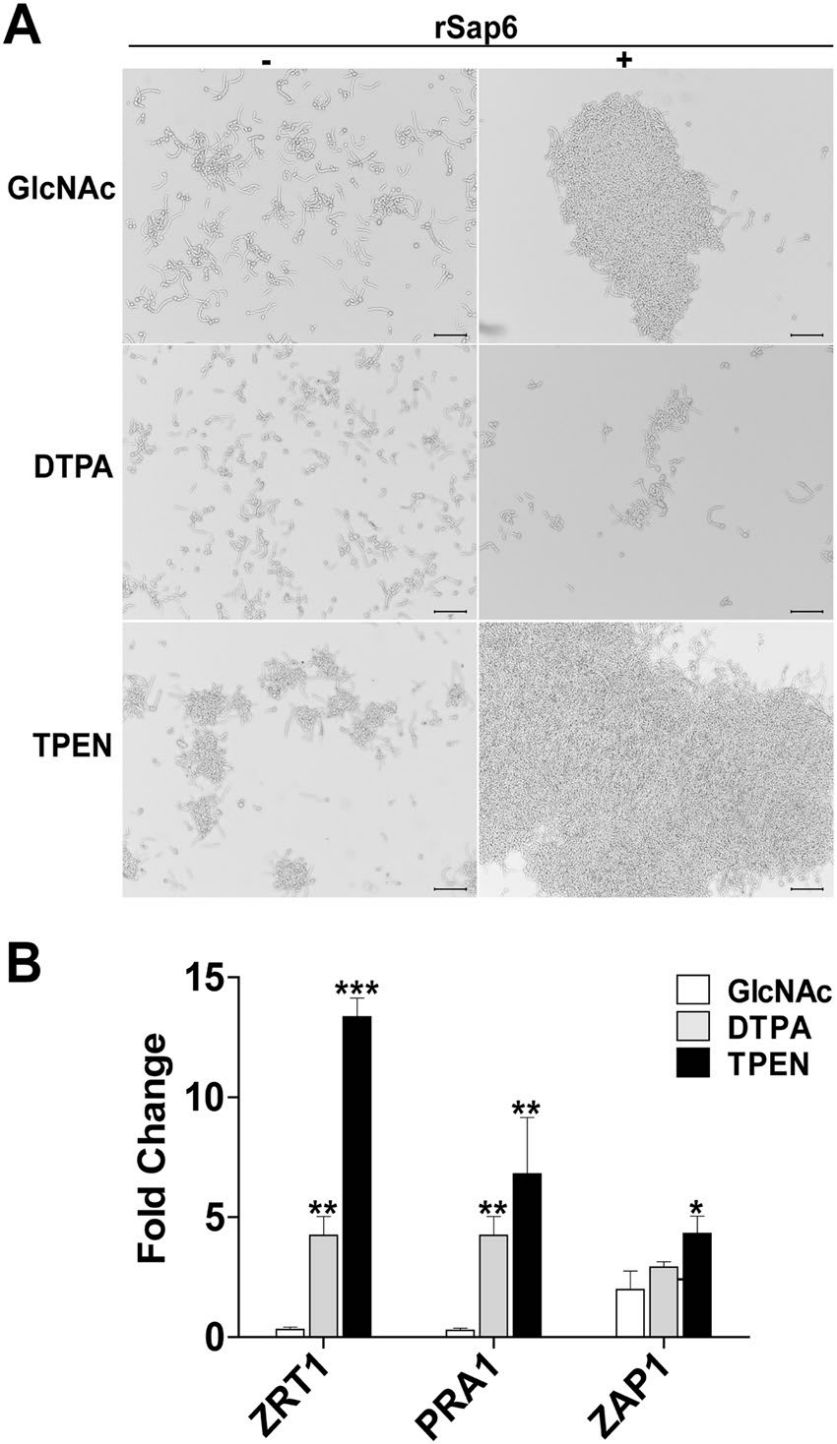
Autoggregation is affected by expression of *ZRT1*, *PRA1*, *ZAP1*, along with extracellular zinc. (**A**) In order to induce increased expression of zinc acquisition genes, cells were incubated with an extracellular (DTPA, 100 µM) or cell permeable (TPEN, 10 µM) zinc chelator and germinated in GlcNAc medium for 3 h at 37 °C, washed and rSap6 (10 µM) was added. (**B**) Expression levels of *ZRT1*, *PRA1* and *ZAP1* in the presence of DTPA and TPEN were assessed by real time PCR using equal amounts of cDNA. DTPA-treated cells had increased expression levels of *ZRT1*, *PRA1*, and *ZAP1* (grey bars); however Sap6-mediated aggregation was nearly abolished in the absence of extracellular zinc (A, middle panel); showing that extracellular zinc is essential to aggregation. TPEN-treated *C*. *albicans* cells with depleted intracellular zinc had the highest transcriptional levels of *ZAP1*, *PRA1* and *ZRT1* (black bars); but addition of Sap6 with restored extracellular zinc resulted in a high aggregation (A, bottom panel) compared to control cells. For each sample real-time PCR was done in triplicate and the experiments were repeated on three independent occassions.

Since the most severe aggregation defect occurred in cells lacking the zinc-responsive transcription factor *ZAP1* and two of its targets (the principal zinc transporter *ZRT1* and extracellular zinc scavenger *PRA1*), we questioned whether increased expression of these genes by zinc-limitation also results in aggregation. To accomplish this, we reduced intracellular or extracellular zinc levels using chelators in order to induce increased expression of these genes and measured aggregation (Fig. 6). *C*. *albicans* cells were germinated in GlcNAc medium (3 h) with or without chelators to selectively deplete either extracellular or intracellular zinc, then Sap6 was added to induce aggregation. Changes in expression levels of *ZRT1*, *PRA1* and *ZAP1* were measured by comparison with yeast cells before treatment. Neither chelator affected the extent of cell germination. Germination of control cells in GlcNAc without chelators (Fig. 6A) did not change *ZRT1* and *PRA1* expression levels significantly, while *ZAP1* expression was increased by 1.5-fold (Fig. 6B). To deplete the media of extracellular zinc, the cell impermeant zinc chelator (DTPA, 100μM) was added to the germination media prior to addition of Sap6.

As expected, DTPA-treated cells maintained in low external zinc media had increased expression levels (by 2–4 fold) of *ZRT1*, *PRA1*, and *ZAP1*; and Sap6-mediated aggregation was nearly abolished (Fig. 6A, middle panel), showing that extracellular zinc is essential to aggregation, independent from the expression of zinc-binding proteins. In contrast, germination of *C*. *albicans* cells with a cell permeable intracellular zinc chelator (TPEN, 10 µM) to selectively deplete intracellular zinc resulted in transcriptional levels of *ZAP1*, *PRA1*, and *ZRT1* that were increased by 4–12 fold over control conditions. Addition of rSap6 to TPEN-treated cells resulted in a robust and significantly higher (by more than 2-fold) aggregative phenotype compared to control cells possibly due to higher *ZRT1* and *PRA1* levels (Fig. 6A). Thus, both higher expression levels of these zinc acquisition genes and the presence of extracellular zinc resulted in increased Sap6 mediated aggregation. This supports our finding that deletion of *ZRT1*, *PRA1*, and *ZAP1* reduce aggregation, and further suggested that these proteins may partner with Sap6 in zinc-mediated aggregation.

### Sap6 participates in *C*. *albicans* zinc accumulation

**Figure 7:**
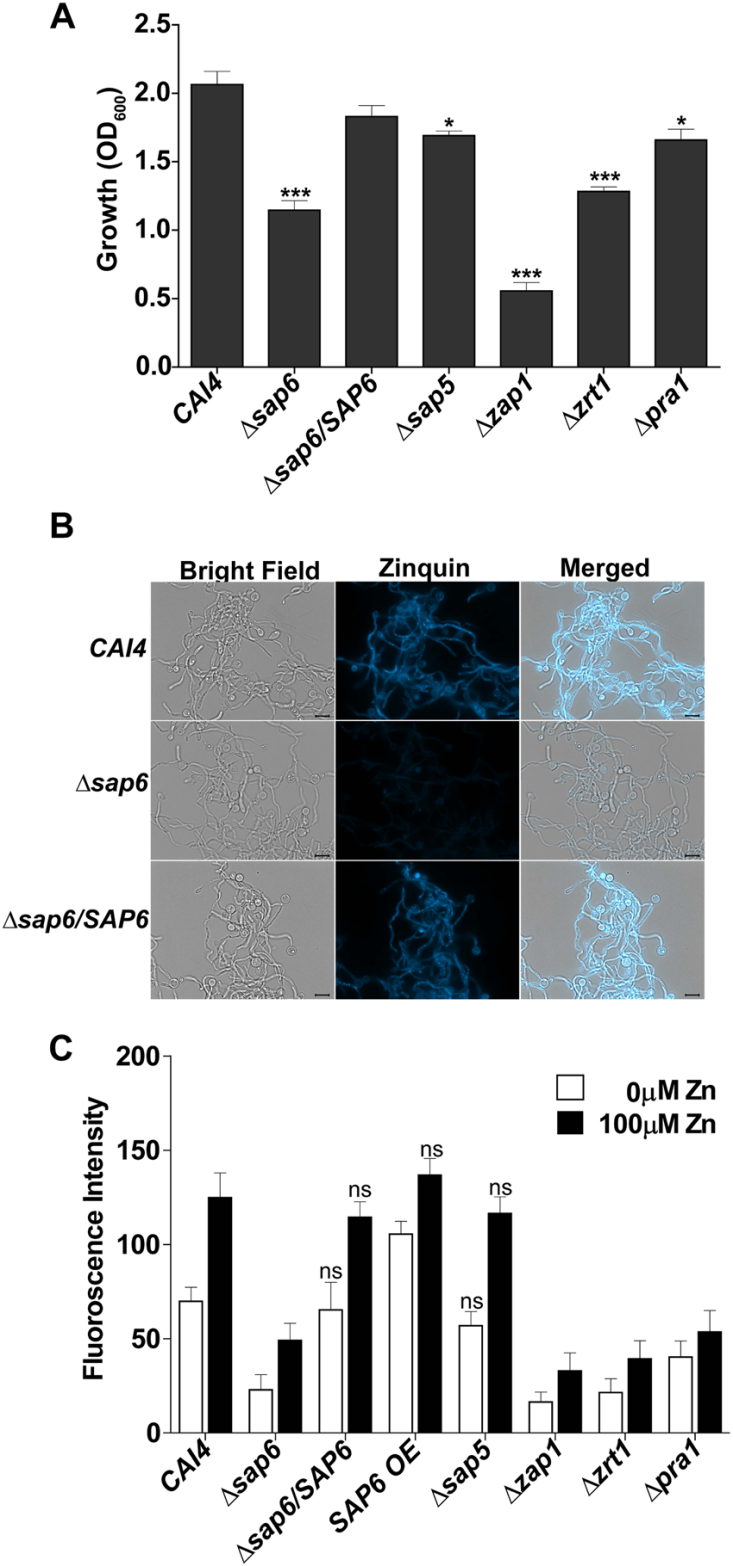
*C*. *albicans* Sap6 deletion results in defective growth in low zinc medium and reduced intracellular zinc accumulation. (**A**) *C*. *albicans* CAI4 and Δ*zap1*, Δ*pra1*, Δ*zrt1*, Δ*sap5*, Δ*sap6*, Δ*sap6*/*SAP6* and *SAP6O/E* cells were inoculated into Low Zinc Medium (LZM) with initial OD_600_ = 0.01, grown for 24 h at 30 °C, and growth measured at OD_600_. *C*. *albicans* Δ*zap1* cells had the largest reduction in growth followed by Δ*zrt1* and Δ*sap6*. Data are averages of three independent experiments. (**B**) CAI4, Δ*sap6* and Δ*sap6*/*SAP6* were grown overnight in LZM, germinated, fixed with paraformaldehyde, and stained with Zinquin (25 µM). Accumulation of intracellular zinc was measured by relative intensity of Zinquin staining. *C*. *albicans* Δ*sap6* strain had decreased Zinquin compared to CAI4 which was reversed upon Δ*sap6*/*SAP6* complementation. (**C**) Cells were grown overnight in LZM, germinated in the presence of 100 µM ZnCl_2_ (black bars) or without added zinc (white bars), and relative intensity of Zinquin fluorescence in hyphal cells measured. *C*. *albicans* Δ*zap1*, Δ*pra1*, Δ*zrt1* and Δ*sap6* all had significantly (P < 0.001) lower Zinquin staining with or without added zinc compared to CAI4. In contrast, *SAP6 O/E* strain had a significant (P < 0.05) increase in Zinquin staining only in low zinc conditions, while the Sap6 revertant (*Δsap6*/*SAP6*) and Δ*sap5* cells were not significantly different than WT cells. Values are mean ± SD of three independent replicates.

Since the presence of extracellular zinc is a central feature of Sap6 aggregation, we hypothesized that Sap6 may sequester zinc at the cell surface in aggregates and aide cells in zinc acquisition. We examined the growth phenotype of *C*. *albicans* Sap mutants (Δ*sap6*, Δ*sap5* and Δ*sap6*/*SAP6* complemented strain) after 24h in zinc-depleted medium (Fig. 7A). *C*. *albicans* Δ*sap6* cells had a significant reduction in growth (44%) compared to WT *CAI4* cells, and this growth defect was reversed in the complement strain Δ*sap6*/*SAP6*. *C*. *albicans* Δ*sap5* cells had only a 18% reduction in growth, showing that Sap5 is less important for growth under low zinc conditions than is Sap6. As expected, *C*. *albicans* ∆*zap1* cells had the most significant reduction in growth (73%), while *C*. *albicans* ∆*zrt1* cells had a similar growth reduction (38%) as Δ*sap6* cells. Interesting, *C*. *albicans* ∆*pra1* cells showed only a mild growth reduction (20%) in zinc-depleted media, similar to that of *C*. *albicans* Δ*sap5* cells (Fig. 7A). Since this data showed that Sap6 is an important contributor to fungal growth in low zinc conditions, we next tested whether Sap6 contributes to intracellular zinc acquisition by direct measurement of cellular zinc levels using the membrane permeable zinc-binding fluorescent dye Zinquin (Fig. 7B).

Cells were grown in low zinc medium for 24 h, then germinated in low zinc (0 µM) or zinc replete (100 µM) media and stained with Zinquin. Strikingly, *C*. *albicans* Δ*sap6* cells had 67% and 60% lower intracellular levels of zinc as WT cells in low and high zinc conditions respectively (Fig. 7B and C). This reduction was between 70–76% for ∆*zap1* and ∆*zrt1* deletion mutants. The Sap6 re-integration strain (Δ*sap6*/*SAP6*) had WT levels of intracellular zinc, while the over-expression strain (*SAP6 O/E*) had significantly increased cellular zinc (51%) compared to WT after growth in low zinc medium. *C*. *albicans* Δ*sap5* cells did not have significantly different intracellular zinc levels than WT cells, whereas ∆*pra1* cells had a 42% reduction in intracellular zinc. These results show that *C*. *albicans* Sap6, but not Sap5, are important proteins for cellular zinc acquisition; and confirm the known roles of Zap1, Zrt1, and Pra1 in zinc uptake.

## Discussion

The classical clinical appearance of oral candidiasis is discrete white plaques that appear microscopically as cellular aggregates of *C*. *albicans* hyphae. Since the oropharyngeal area is a relatively nutrient poor environment^4^, community interactions through cellular aggregation are likely to be important for *C*. *albicans* to acquire essential carbon, nitrogen and metals from its surroundings. Amyloid forming sequences are common within fungal adhesion proteins^29^. Cellular aggregation is a common function of amyloids, as shown by amyloid containing *C*. *albicans* Als proteins^22^, and other cell surface adhesins such as Hwp1/2, Ece1, Rbt5 and Sap3^20^. Metal ion binding by amyloids is a fundamental property of these structures that causes formation of oligomeric or polymeric complexes. For example, Aβ40 amyloid can bind to Zn^2+^ ions with high affinity due to presence of amyloid metal binding sites resulting in oligomer formation^24, 25^. In this study, we computationally and functionally identified four amyloid regions within Sap6 that bind Zn^2+^, and this binding increased autoaggregation. However, amyloid forming regions can interact both with self (same protein) as well as non-self (other non-amyloid proteins) to induce autoaggregation, in addition to promoting adhesion between *C*. *albicans* and host cells^22^. Among non-self proteins, we have identified *C*. *albicans* Zap1 regulated proteins to be key proteins that mediate aggregation through Sap6. In addition, it is possible that amyloids present on bacterial^32^ and host cell^33, 34^ surfaces, may be involved in *C*. *albicans* adhesion to bacterial or host tissues. It is also important to recognize that within the *C*. *albicans* Sap gene family, Saps 4–6 share close sequence and functional similarities^7^, so that similar amyloid domains in Sap4 or Sap5 may also contribute to cellular aggregation although we found that Sap5 does not contribute to zinc acquisition.

During oral candidiasis, metal (mainly zinc and iron) limitation genes, along with Sap6, were highly up-regulated during infection, suggesting the importance of metal acquisition for virulence^4^. The best-described *C*. *albicans* zinc-binding protein is Pra1, whose levels are only slightly increased in OPC or in late-phase of RHE infections^4, 35^. *PRA1* is expressed under conditions of zinc depletion, and following secretion and zinc binding, it re-associates with the hyphal surface where it presumably releases zinc to Zrt1 cell surface transporters^14, 36^. This Pra1 zincophore system is regulated by *ZAP1* (being induced only in low zinc)^37^ and Pra1 expression and secretion is limited to neutral to alkaline pH^38, 39^. Since the oral environment is slightly acidic^40^, it is possible that Pra1 function is limited in this environment and may explain its low expression levels in OPC.

Sap6 may be an alternative zincophore system in the oral and GI environment as it is secreted under a wider pH range (5.0–7.0)^6, 41^, and its expression may be regulated by additional factors beyond zinc limitation. Although *ZAP1* activates zinc acquisition genes, it also plays an important role in cell adhesion by regulating expression of cell surface adhesion proteins along with Sap6^16^. Among *C*. *albicans* cells with aggregation defects (Fig. 5), we identified Pra1, Zrt1, Ece1, Hwp1, Hyr1, Csa1 and Csa2 proteins that are regulated by both Rim101 and Zap1 depending upon signals from the local environment. Rim101 also regulates genes expressed during zinc and iron limitation at higher pH, along with Sap5 and Sap6^40^; and in *Saccharomyces cerevisiae* Rim101 and Zap1 can physically interact with each other^42^. Thus, *C*. *albicans* transcriptional regulation of zinc acquisition and surface proteins that bind with Sap6 to promote aggregation may be co-regulated by both *RIM101* and *ZAP1*.

Our data show that Sap6 has dual functions as both a secretory molecule that induces self-aggregation through amyloid and zinc binding, but also acts as another fungal zincophore system as shown by the requirement of Sap6 for growth in low zinc conditions and in zinc uptake (Fig. 7). Thus, zinc participates in Sap6 amyloid-dependent autoaggregation, which may also be a means of single cell as well as community acquisition of zinc. Furthermore, the protease activity of Sap6, that we previously showed is not needed for autoaggregation^3^, may function to release zinc through degradation of bound metalloproteins within fungal aggregates. Autoaggregation may therefore be a mechanism by which *C*. *albicans* sequesters free zinc or other zinc-containing proteins in the process of zinc acquisition.

**Figure 8:**
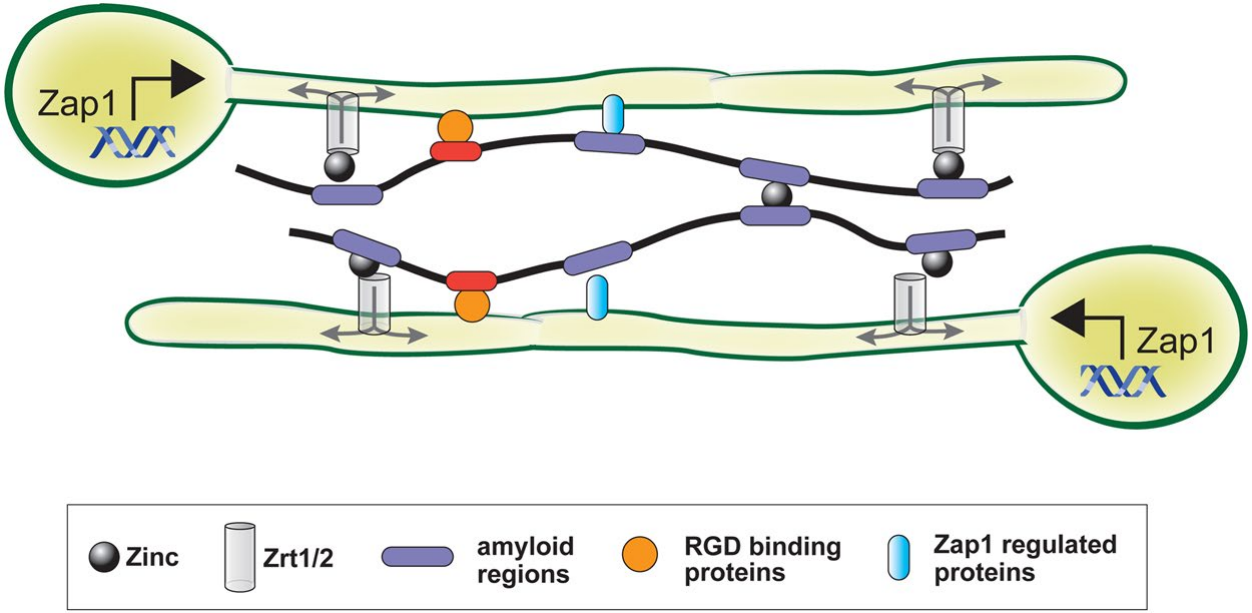
Model of Sap6 mediated cell-cell aggregation. *C*. *albicans* Sap6 contains amyloid regions (purple ovals) that function in autoaggregation by binding with hyphal surface Zap1 regulated proteins (blue ovals) and by binding with other amyloid regions mediated by zinc. Amyloid binding of zinc may also function to transfer zinc to fungal zinc transporters Zrt1/2 for uptake under zinc limiting environments. Sap6 RGD binding domains (red ovals) also binds cell surface RGD adhesins (gold circles) that contribute to autoaggregation.

While Sap6 amyloids account for more than half of autoaggregation, Sap6 RGD regions also contribute to aggregation. It is possible that Sap6 RGD domains may function more in cell-host binding to tether cell aggregates to host tissues; while amyloid regions mediate both autoaggregation and zinc acquisition. Our results suggest a model (Fig. 8) whereby *ZAP1* and perhaps *RIM101* are master regulators for expression of Sap6 and other cell surface proteins that mediate autoaggregation through amyloid domains of Sap6. These self-aggregates then serve to sequester zinc and, along with Pra1 and the zinc transporter Zrt1, serve to maintain zinc homeostasis within *C*. *albicans* aggregated communities. Our work shows that *C*. *albicans* Sap6 has multiple functions for autoaggregation and zinc uptake in addition to its previously described protease activity. Targeting Sap6 mediated aggregation either by metal chelation or direct inhibition with small molecules could provide a new therapeutic avenue for combating fungal infections, as has been shown in treatment of pulmonary aspergillosis^43^.

## Material and Methods

### Strains, reagents and growth conditions

*C*. *albicans* strains used are listed in Table 1. A library set of *C*. *albicans* homozygous mutants was obtained via The Fungal Genetics Stock Center (FGSC)^44^. *C*. *albicans* complementation strain (*Δsap6*/*SAP6*) was constructed by introduction of one copy of the *SAP6* gene at the RPS10 locus in *Δsap6* cells. Complementation strains of *ZRT1* (Δ*zrt1*/*ZRT1*) and *PRA1* (Δ*pra1*/*PRA1*) genes were constructed by PCR and homologous recombination using vector pSN105 as described earlier^45^. For most experiments, a single colony was inoculated in YPD broth, grown overnight at 30 °C with shaking at 220 rpm to reach OD_600_ = 3.0. To germinate cells, overnight cultures were washed twice in Phosphate Buffered Saline (PBS, 10 mM), diluted to OD_600_ = 0.3 in pre- warmed (37 °C) Yeast Nitrogen Base (YNB) medium containing 1.25% N-acetyl D-glucosamine (GlcNAc) (Sigma Aldrich) and incubated for 3 h at 37 °C. For zinc limiting experiments, cells were cultured for 24 h at 37 °C in yeast nitrogen base without Zn^+^ (MP Biomedicals). For experiments using divalent metals, ZnCl_2_, FeCl_3_ or CuSO_4_ (Fisher Scientific) were added to the medium to final concentration of 10 µM. Hyphal formation of all mutants under all conditions was verified microscopically over several fields to include at least 200 cells, and quantified to ensure that 90% percent germination was achieved. Recombinant rSap6 and rSap6_ΔRGD_ were expressed and purified from culture supernatants of recombinant *Pichia pastoris* strains as described^3^. Stock solutions (1 mM) of Congo red and Thioflavin T (Sigma Aldrich) was syringe filtered using 0.22 µm filters immediately prior to use.

**Table 1:** *C*. *albicans* strains used in this study.

Strain	Genotype	Reference
CAI4	*Δura3::imm434 Δura3::imm434 RPS1 Δrps1::CIp10-URA3*	3
*Δsap6*	*Δsap6::hisG*/*Δsap6::hisG-URA3-hisG*	3
*Δsap6*/*SAP6*	*Δsap6::hisG*/*Δsap6::hisG-URA3-hisG:: RPS1-SAP6-URA3*	This study
*Δzrt1*/*ZRT1*	*his1Δ*/*his1Δ*, *leu2Δ*/*leu2Δ::*ZRT1-C. dubliniensisARG*4*, *arg4Δ*/*arg4Δ4*, *URA3*/*ura3Δ::imm434*, *IRO1*/*iro1Δ::imm434 Δzrt1::C*. *dubliniensisHIS1*/*Δzrt1:::C*. *maltosaLEU2*	This Study
*Δpra1*/*PRA1*	*his1Δ*/*his1Δ*, *leu2Δ*/*leu2Δ::*PRA1-C. dubliniensisARG*4*, *arg4Δ*/*arg4Δ4*, *URA3*/*ura3Δ::imm434*, *IRO1*/*iro1Δ::imm434 Δpra1::C*. *dubliniensisHIS1*/*Δpra1:::C*. *maltosaLEU2*	This Study
*SAP6 OE*	*Δura3::imm434 Δura3::imm434 RPS1 Δrps1::CIp10-SAP6-URA3*	3
*Δals1*/*Δals3*	*als1::hisG als1::hisG als3::dpl::200 als3::dpl200*	56
*Δint1*	*int1Δ::hisG*/*int1Δ::hisG-URA3-hisG*	57
CJN1193 (ZAP1 complement)	*∆ura3::imm434 arg4::hisG his1::hisG::pHIS1-ZAP1 zap1::ARG4 ∆ura3::imm434 arg4::hisG his1::hisG zap1::URA3*	37
CJN1623(PRA1O/E)	*∆ura3::imm434 arg4::hisG his1::hisG::pHIS1 zap1::ARG4 PRA1::pAgTEF1-NAT1-AgTEF1UTR-TDH3-PRA1 ∆ura3::imm434 arg4::hisG his1::hisG zap1::URA3 PRA1*	37
CJN1651(ZRT1O/E)	*∆ura3::imm434 arg4::hisG his1::hisG::pHIS1 zap1::ARG4 ZRT1::pAgTEF1-NAT1-AgTEF1UTR-TDH3-ZRT1 ∆ura3::mm434 arg4::hisG his1::hisG zap1::URA3 ZRT1*	37
JFY348(ZAP1O/E)	*ura3∆::•imm434 ARG4:URA3:arg4::hisG his1::hisG::pHIS1 ZAP1:pAgTEF1-NAT1-AgTEF1UTR-TDH3-ZAP1 ura3∆::imm434arg4::his1 his1::hisG ZAP1*	16
SF1280 (ECM331 complement)	*SC5314 – ECM331::pAgTEF1-NAT1-AgTEF1UTR-TDH3-ECM331*	58
*Δals5*	See reference	45
*Δals9*	See reference	45
*Δcsa1*	See reference	45
*Δcsa2*	See reference	45
*Δece1*	See reference	45
*Δecm331*	See reference	58
*Δhwp1*	See reference	59
*Δhwp2*	See reference	59
*Δhyr1*	See reference	45
*Δhyr3*	See reference	45
*Δiff4*	See reference	45
*Δpga10*	See reference	45
*Δpga13*	See reference	45
*Δpga17*	See reference	60
*Δpga18*	See reference	45
*Δpga49*	See reference	45
*Δpga60*	See reference	45
*Δsap5*	See reference	45
*Δscw4*	See reference	45
*Δspr1*	See reference	45
*Δsod6*	See reference	45
*Δpra1*	See reference	45
*Δzrt1*	See reference	45
*Δzap1*	See reference	45

### *In silico* analysis of amylogenic regions

Protein sequence of Sap6 was obtained from the *Candida* Genome Database and was analyzed using AmylPred2 (http://aias.biol.uoa.gr/AMYLPRED2)^46, 47^. Briefly, AmylPred2 uses a consensus of overlaps from 5 of 11 different methods known, or specifically designed, to predict features related to the formation of amyloid fibrils. Each predicted amyloidogenic regions from Sap6 was further confirmed using TANGO (http://tango.crg.es/) and PASTA 2.0(http://protein.bio.unipd.it/pasta2/)^48, 49^.

### Modeling of three-dimensional structure and zinc binding sites

Homology modeling server Phyre2 (http://www.sbg.bio.ic.ac.uk/phyre2) was used to predict Sap6 three-dimensional homology structure^50^. The predicted Sap6 protein model was validated using SAVES server (http://services.mbi.ucla.edu/SAVES/). Visualization and molecular graphics of different regions on Sap6 structure were performed using UCSF Chimera^51^. For prediction of metal binding sites, Metal Ion-Binding (MIB) site prediction server (http://bioinfo.cmu.edu.tw/MIB/) was used, which predicts metal binding sites using protein three-dimensional structure^52^. Sequence based ligand binding prediction software IonCom (http://zhanglab.ccmb.med.umich.edu/IonCom) was used to confirm predicted metal binding sites^53^.

### Congo red spectroscopic amyloid assay

Four predicted amyloidogenic regions of Sap6 (P1–P4) and a scrambled version of the P2 (sP2) peptide were synthesized by Genemed Synthesis (San Antonio, Texas) with a final purity of >95%. To test amyloid formation, rSap6, sP2 and P1–P4 peptides were diluted in deionized water (10 µM final concentration) and incubated at 37 °C for 4 h. Freshly prepared Congo red (20 μM) was added to P1–P4 peptides (final reaction volume 200 µl) and incubated for 20 min at 25 °C before measuring absorbance spectrum (400 nm–700 nm)^29^. Congo red alone prepared in deionized water was used as reference spectrum whereas absorbance spectrum of rSap6 or peptides (sP2, P1–P4) alone were used as control for solution turbidity caused by aggregate formation. The experiment was performed using black clear bottom 96 well plate and the absorbance was measured with a FlexStation3 multimode microplate reader (Molecular Devices). The absorbance spectrum of the protein/peptide alone were subtracted from Congo red-peptide samples to correct turbidity. The normalized spectrum was plotted along with Congo red reference spectrum using GraphPad software.

### Thioflavin T amyloid assay

To study the role of zinc on amyloid formation, a fluorescence Thioflavin T(ThT) assay was performed in a 96-well black clear bottom microtiter plate as described^29^. In experiments with zinc, ZnCl_2_ (10 µM–500 µM) was added to 10 µM peptides (sP2 and P1–P4) and rSap6 and incubated for 4 h at 37 °C and for additional 1 h at 25 °C in dark. Peptides (10 µM) and rSap6 (10 µM) zinc complex were mixed to a final volume of 200 μl with 20 μM ThT solution (freshly made from 1 mM ThT stock). Fluorescence measurements were made using a FlexStation3 multimode microplate recording spectra from 440–560 nm (Ex = 438 nm; Em = 495 nm) using a 475 nm cutoff with a 5 nm slit for both excitation and emission. The spectrum of ThT alone was used as negative control. The fluorescence spectrum of test peptide or Sap6 was plotted after subtracting control ThT spectrum using GraphPad software.

### Cell aggregation assays

Cell aggregation assays were performed as described^3^. Briefly, *C*. *albicans* cells were germinated for 3 h at 37 °C, harvested by centrifugation (200 × g) for 5 min, washed twice in 10 mM PBS buffer and adjusted to 10^6^ cells/ml. Germinated cells (10^6^/ml) with or without rSap6 (10 µM), or P1–P4 and sP2 peptides (10 µM) were incubated for 15 min to allow aggregation, then aggregates were visualized with a Zeiss AxioScope A.1 microscope and aggregate size (average diameter of aggregates from 20 independent fields) was determined using Image J^54^.

To study interactions between Sap6 and divalent ions, rSap6 and ZnCl_2_, FeCl_3_ or CuSO_4_ were mixed in equimolar ratios for 1 h at 37 °C; then added to germinated cells to determine cellular aggregation. Addition of metals alone (ZnCl_2_, FeCl_3_ or CuSO_4_ 10 µM) to germinated cells was used as control. For investigating the role of Congo red on cell aggregation, pre-germinated *C*. *albicans* cells were incubated with Congo red (30 µM) after adding rSap6 or rSap6_ΔRGD_. Statistical analyses were conducted with one-way ANOVA followed by Tukey multi comparison test using GraphPad software.

### Determination of Sap6 binding with zinc using zincon

Sap6 binding to zinc was determined by competition binding with the colorimetric Zn chelator zincon (2-carboxy-2′- hydroxy-5′-(sulfoformazyl)benzene, Sigma Aldrich) as described with the following modifications^27^. A 1.5-mL solution containing 20 µM Zincon and 10 µM rSap6 was prepared in a quartz cuvette (75 mM HEPES, 100 mM NaCl, pH 7.4). Bovine serum albumin (BSA, Sigma Aldrich) (10 µM) was used as a positive control whereas the scrambled peptide sP2 was served as negative control. Zincon forms a 1:1 complex with Zn^2+^, which absorbs at 621 nm. The solution was titrated with 0–5 equivalents of Zn^2+^ ions (2 µL of 1 mM ZnCl_2_ aqueous solution per addition) at 25 °C. The samples were allowed to equilibrate for 10 min after each Zn^2+^ addition and changes in absorbance at 621 nm was plotted as the [Zn^2+^]/[Substrate] ratio. The apparent binding constant (K_app_) for Sap6 was determined by titrating rSap6 (0–50 µM) against a Zincon-Zn complex, using a Zincon K_d_ value of 10 µM^27^ and calculated by the equation:

$$\frac{[Zn\ast rSap6][Zincon]}{[rSap6][Zn\ast Zincon]}=\frac{Kapp(Zn\ast rSap6)}{Kd(Zn\ast Zincon)}$$

### Zinc limitation using chelators

For zinc chelation, the membrane permeable chelator N, N, N′, N′-Tetrakis (2-pyridylmethyl) ethylenediamine (TPEN) and the membrane impermeable chelator diethylenetriaminepentaacetic acid (DTPA) were used. The cells were allowed to germinate in YNB + 1.25% GlcNAc medium containing either DTPA (100 µM) or TPEN (10 µM) for 3 h at 37 °C, then washed twice with PBS before addition of rSap6 (10 µM).

### Gene expression analyses

*C*. *albicans* WT cells germinated in YNB + 1.25%GlcNAc medium with DTPA (100 µM and TPEN (10 µM) for 3 h at 37 °C were harvested and the cell pellet was used for RNA isolation as described previously^3^. Following isolation, RNA purity and concentrations were determined using a Nanodrop 100 and the integrity of total RNA was checked on formaldehyde gel. The total cDNA was synthesized for each sample using an iScript cDNA synthesis kit following the manufacturer’s instructions, with equal amounts of RNA (2 µg in a 20 µl reaction mixture).

To quantify gene expression of ZAP*1* (forward ACATTATCGGGTTCATTCAG, reverse-TACAAACCAATGGTCTTTCC) *ZRT1* (forward-CAACCAATACAAACAACCTTCCT reverse-CAACACCAGCATGGAAATGACA and *PRA1* (Forward-GTTGTCGGTGCTGACAAATCA, reverse CGGAGCATAGTTGGGATAAGTATCT). The cDNA of each sample was used to amplify transcripts using CFX-96 Touch Real Time PCR system (Bio-Rad) as described previously^55^. Fluorescent data were collected and analyzed with iCycler iQ software. The standard curve method was employed for relative quantification using *GAPDH* (forward AAGAGTTGCTTTGGGCAGAA, reverse GTCGTCACCAGAAGCAGTGA) and *ACT1* (forward TCGGTGACGAAGCTCAATCCAAGA, reverse CAATGGATGGACCACTTTCGTCGT) as housekeeping gene controls. Relative quantities of the mRNAs for the genes of interest and housekeeping genes were calculated from the corresponding standard curves. Error bars represent the standard difference between replicates, and results are the mean of at least three independent biological replicates.

### Binding of rSap6 to *C*. *albicans*

rSap6 was labeled with fluorescein inisothiocyanate (FITC) (Sigma-Aldrich) as previously described^3^. Germinated cells were fixed with 4% paraformaldehyde for 1 h. F-rSap6 (10 µM) was added to cells 1 h at 37 °C in the dark, washed twice with 10 mM PBS, and binding of F-rSap6 was documented with an inverted fluorescence microscope (Zeiss Axio Observer.Z1) at 63 X magnification.

### Intracellular zinc quantitation using Zinquin

Cells were grown in low zinc medium for 24 h, then germinated overnight with 1.25% N-acetyl D-glucosamine (GlcNAc) in low zinc medium with or without 100 µM ZnCl_2._ Hyphal cells were washed twice with Chelex-100 treated 10 mM PBS to remove any metal contamination, and fixed with 4% paraformaldehyde. Fixed hyphal cells were washed twice, re-suspended in Chelex-100 treated 10 mM PBS (10^4^ cells/ml), and stained with 25 µM Zinquin ethyl ester (Santa Cruz Biotechnology) for 1 h at 30 °C. For intracellular Zinquin visualization under microscope, inverted Axio Observer.Z1 using a DAPI filter was used. For quantification of Zinquin fluorescence, stained cells were transferred to 96-well black clear bottom microtiter plates and fluorescence intensity was measured at 370 nm (excitation) and 490 nm (emission) using a FlexStation3 multimode microplate reader (Molecular Devices). Fluorescence intensities (1000 hyphal cells/well) were measured using unstained cells as experimental control. Data are the mean ± SD of at least three independent measurements. Statistical analyses were conducted using two-way ANOVA followed by Bonferroni post-test using GraphPad software.
